# Constructing Superhydrophobic Surface on Copper Substrate with Dealloying-Forming and Solution-Immersion Method

**DOI:** 10.3390/ma15144816

**Published:** 2022-07-10

**Authors:** Hui Li, Yannan Sun, Zhe Wang, Shiyi Wang

**Affiliations:** Guangdong Provincial Key Laboratory of Micro/Nano Optomechatronics Engineering, College of Mechatronics and Control Engineering, Shenzhen University, Nanhai Ave 3688, Shenzhen 518060, China; sunyannan816@163.com (Y.S.); wangzheszu@163.com (Z.W.); wangshiyi1023@126.com (S.W.)

**Keywords:** superhydrophobic, dealloying, nanostructures, copper stearate, contact angle

## Abstract

In this study, a superhydrophobic surface was constructed on a copper substrate through dealloying-forming and solution-immersion methods. The dealloying process for nanostructures on a copper surface involved the electrodeposition of zinc atoms, and the thermal alloying and chemical dealloying of zinc atoms. Then, a dealloyed copper surface was subsequently modified with low-surface-energy copper stearate to produce a superhydrophobic surface. Scanning electron microscopy, X-ray diffractometry, and Fourier transform infrared spectrometry were employed to characterize the morphological features and composition components of the surface in the fabrication process. The static contact angles of the copper surfaces were compared and evaluated based on various fabrication parameters, including electric current density, corrosive solution concentration, and nanostructures. The results indicated that a leaf-like copper stearate could be constructed through immersing a dealloyed copper plate into a 0.005 mol/L ethanol solution of stearic acid for 5 min. Nanostructures provided more attachment areas on the copper surface to facilitate the formation of copper stearate. The resulting as-prepared surface presented excellent superhydrophobic properties with a contact angle of over 156.5°, and showed the potential properties of non-sticking, self-cleaning, anti-corrosion, and stability. This study provides an efficient approach to fabricate superhydrophobic surfaces for engineering copper metals.

## 1. Introduction

The phenomena of wettability and adhesiveness between water and solid surfaces have been the topic of much research, owing to their wide range of practical applications to daily life [1,2,3]. Superhydrophobicity with water contact angles greater than 150° can be used in special fields, such as self-cleaning [4], anticorrosion [5], anti-icing [6,7], oil–water separation [8], etc. Hierarchical structures and low surface free energy are key elements in superhydrophobic surfaces. To date, many strategies have been employed to manufacture superhydrophobic surfaces, including electrochemical deposition [9], solution-immersion [10], chemical etching [11], self-assembly [12], sol-gel [13], etc.

As a common industrial material, copper is widely employed due to its excellent mechanical workability, high electrical, thermal conductivity, etc. [14]. Many studies have been performed on the construction of hierarchical structures on copper to produce superhydrophobic surfaces. Chu et al. [15] fabricated micro- and nano-structures on a copper surface via the “two simultaneous chemical reactions” method. Through processing the metal surfaces with deposition and etching, the contact angle of the treated surface exceeded 157° with moss-like hierarchical structures. He et al. [16] fabricated a superhydrophobic coating on a copper substrate by electrodeposition and thermal annealing. The resulting surface exhibited excellent superhydrophobicity, with a water contact angle as high as 170°. Xie et al. [17] produced hierarchical nanospheres on a silicone rubber surface using direct laser etching. The resulted surface exhibited a contact angle of 154 ± 3° and maintained its superhydrophobic state under a dynamic pressure of 1960.2 Pa. However, there are inevitable issues for these fabrication methods, including an expensive experimental setup, complicated technical procedures, and toxic fluorine-containing reagents, all of which hinder the ability to use these products in real-life applications.

However, a more convenient approach, the solution-immersion method, has gained widespread acceptance to construct superhydrophobic substrates on various materials, including copper [18], magnesium alloy [19], steel [20], etc. The frequently used solutions were a fatty acid solution [21] and stearic acid [22]. Jiang et al. [23] first developed a superhydrophobic surface with an ethanol solution with *n*-tetradecanoic acid (0.5 M) as the electrolyte. With flowerlike clusters gradually forming on the surface, the contact angle increased to 153.9° after an immersion time exceeding 3 h. However, the manufacturing process was time-consuming. To shorten the time required for the immersion surface to reach superhydrophobicity, nanostructures can be fabricated on the target metal surface in advance. Feng et al. [24] fabricated zinc nano-coatings on carbon steel through an electrodeposition method, then the samples were immersed into a 0.05 mol/L stearic acid solution for modification. They found that the surface became superhydrophobic after 20 min. Rezayi and Entezari [25] deposited wire-like ZnO particles on an iron surface to increase the surface roughness, and subsequently, stearic acid was utilized for wettability modification. The water contact angle reached 159.89° with a stearic acid concentration of 0.01 g/cm^3^.

In this study, copper substrates with nanostructures on the surface were prepared through a dealloying method. Then, a superhydrophobic surface was fabricated by directly immersing the sample into an ethanol solution of stearic acid. The manufacturing process and wettability of the copper surface were evaluated via scanning electron microscopy (SEM), X-ray diffractometry (XRD), Fourier transform infrared spectrometry (FTIR), and contact-angle measurement. SEM images revealed the micro- and nano-hierarchical morphologies, and FTIR confirmed the presence of stearic copper. The wettability and practical applications of the as-prepared superhydrophobic surface were investigated in detail. We think that the high efficiency and cost-effectiveness of the fabrication process in this study show the advantages of our method compared with previous methods.

## 2. Experimental Section

### 2.1. Materials

Copper substrates (purchased form Kesheng Technology Co., Suzhou, China) with a purity of >99.9% were employed, while the dimensions of all samples were 20 × 20 × 2 mm^3^. Zinc (purchased from Jinjia Metal Co., Hebei, China) with a purity of >99.8% was used in the electrodeposition process. Stearic acid and ethanol were purchased from Aladdin. A H_2_SO_4_ solution was purchased from Bolaien Technology Co., Shenzhen, China. All the reagents were of analytical grade throughout the experiments.

### 2.2. Preparation of Superhydrophobic Surface

**Figure 1:**
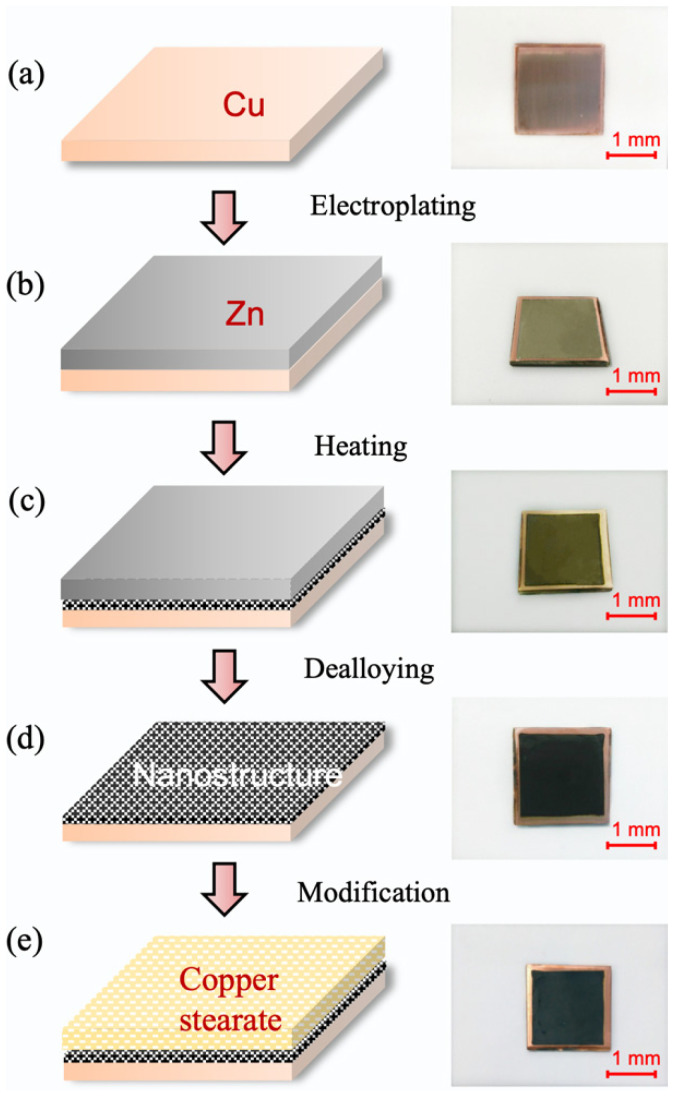
Schematic preparation procedure and photographs of superhydrophobic copper surface at each step, (**a**) preprocessing; (**b**) electroplating; (**c**) heating; (**d**) dealloying; and (**e**) modification.

Before the experiment, the copper substrates were polished with SiC paper of successively finer grits down to 2000 grit, ultrasonically degreased in deionized water for 5 min, degreased in a 120 g/L NaOH solution for 2 min, followed by an oxide removal by immersion into the H_2_SO_4_ solution. Then, the substrates were rinsed with deionized water and dried by nitrogen gas at room temperature, as shown in Figure 1a.

During the electrodeposition process, zinc was used as the positive electrode, while the copper substrate was used as the negative electrode in the zinc electroplating bath, which contained a ZnCl_2_ solution. The current was controlled by a DC power system (PWS4323, Tektronix, Beaverton, OR, USA). Then, the zinc layer was coated onto the cleaned copper substrate under a direct current condition, as shown in Figure 1b. The electric current density was maintained between 10 and 35 mA/cm^2^, while electroplating took 20 min. Then, the sample was heated with a temperature of about 150 °C in a vacuum sintering furnace under a protective environment with hydrogen gas. The pressure was maintained at about 0.3 MPa, and the heating time was between 1 and 3 h. The appearance of the sample after thermal treatment is illustrated in Figure 1c. Lastly, to remove the zinc layer, the dealloying process was conducted in an acid solution, as shown in Figure 1d. Figure 1e presents the image of the copper surface after modification treatment. Through immersion into the ethanol solution of stearic acid (0.005 mol/L), the dealloyed surface was modified within 5 min to 12 h at room temperature, then rinsed with acetone and deionized water, and finally dried with nitrogen gas.

### 2.3. Characterization of the Prepared Samples

The morphologies of the prepared surfaces were characterized with field-emission scanning electron microscopy (SEM, 1530 VP, LEO, München, Germany). The component analysis was conducted through an X-ray diffractometer (XRD, D8 ADVANCE, Bruker Optics, Germany) and a Fourier transform infrared spectrometer (FTIR, VERTEX 33, Bruker Optics, Germany). The static water contact angle was measured with a contact angle meter in an ambient environment. The volume of the deionized water droplet was 4 μL, and the room temperature was 25 °C. To reduce test errors, the value was calculated as the average of five different positions on the surface.

## 3. Results and Discussion

### 3.1. Fabrication Process of the Superhydrophobic Surface

**Figure 2:**
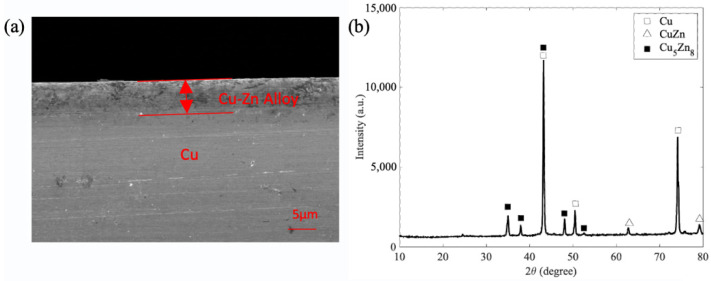
Characterization of the copper surface after dealloying for 30 min, (**a**) SEM image and (**b**) XRD pattern.

Figure 2 shows the SEM image and XRD pattern of the copper surface after thermal treatment. To demonstrate the formation of a Cu-Zn alloy layer on the copper surface, an SEM image with a cross-sectional view is shown in Figure 2a. The zinc elements diffused into the copper during thermal treatment. Figure 2b shows the component analysis of the surface using XRD. The XRD pattern of the Cu-Zn alloy substrate after thermal treatment indicated that there were peaks of pure Cu, compared with the results of JCPDS Card No. 04-0836. The other peaks could be well-indexed to CuZn (JCPDS Card No. 02-1231) and Cu_5_Zn_8_ (JCPDS Card No. 25-1228). Based on the intensity, Cu_5_Zn_8_ presented a higher Zn content in the 2θ range of 10°–80°.

**Figure 3:**
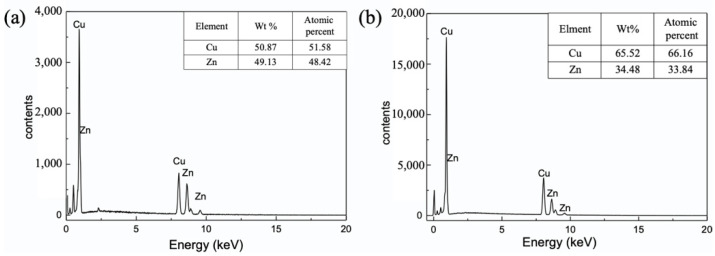
EDS patterns of the copper surfaces dealloyed by a H_2_SO_4_ solution for (**a**) 5 min and (**b**) 30 min.

After chemical corrosion in an acid solution for 30 min, the color of the surface turned black due to its high specific surface area, as shown in Figure 1d. This indicated that the alloying and dealloying processes of the copper substrate and zinc layer resulted in the construction of three-dimensional nanostructures. Figure 3 shows the EDS patterns of the copper surface after the dealloying process. When the dealloying time was 5 min, the surface mainly consisted of Cu and Zn elements. The weight of the copper content was 50.87%, while that of the zinc content was 49.13%, as shown in Figure 3a. Subsequently, when the dealloying time was raised to 30 min, the copper content increased to 65.53%, and the zinc content dropped to 34.48%, as shown in Figure 3b. This phenomenon was caused by the reaction between the zinc and corrosion solution H_2_SO_4_. However, the zinc content was not completely depleted after the 30 min dealloying treatment.

**Figure 4:**
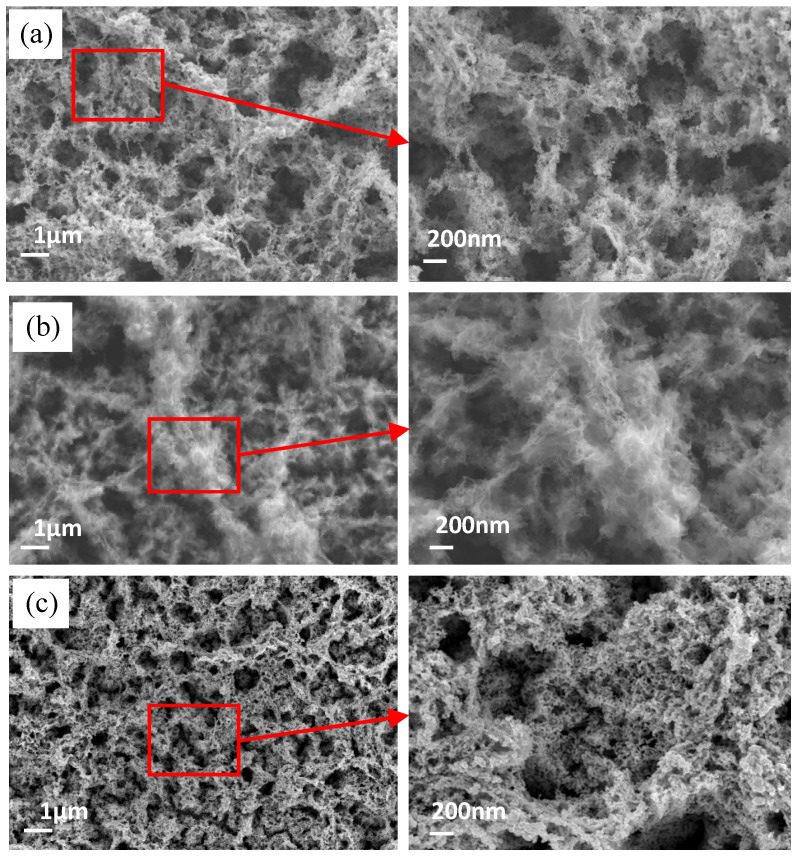
SEM images of the surface after dealloying for different times: (**a**) 5 min; (**b**) 10 min; (**c**) 30 min.

Figure 4 shows SEM images of the surface topography after the dealloying treatment within different timeframes. After only 5 min of corrosion, the nanostructures appeared on the surface, as shown in Figure 4a. The sizes of the different nanopores varied, ranging from 10 to 800 nm. Over the course of the 10 min dealloying process, the micro-skeletons began to dissolve, as shown in Figure 4b. However, when the dealloying time was extended to 30 min, the copper surface possessed more continuous nanostructures with pore sizes of under approximately 500 nm.

**Figure 5:**
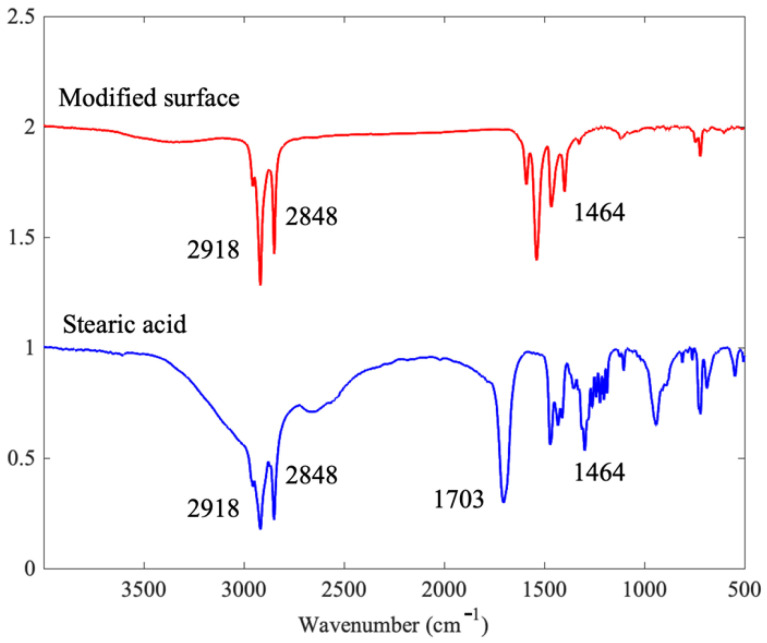
FTIR spectra of stearic acid and stearic copper surface after modification treatment.

After the modification process in an ethanol solution of stearic acid, the chemical composition Cu[CH_3_(CH_2_)_16_COO]_2_ was formed and attached to the nanostructure surface. FTIR spectra were used to analyze the component characterization of the copper surface after modification, as shown in Figure 5. The peak of 1703 cm^−1^ in the stearic acid spectrum disappeared after the reaction, while a new peak was observed at the wavenumber of 1589 cm^−1^. We attribute this behavior to the possible bonding mechanism of coordinated COO moieties. The peaks between 3000 and 2600 cm^−1^, as well as the peaks around 1300 cm^−1^, corresponded to the CH bonds in the sum of the methyl and methylene groups. The overall reactions for the modification treatment were formulated with the following equations,

(1) $$2\mathrm{Cu}+O_{2}+4H^{+}\to 2{\mathrm{Cu}}^{2+}+2H_{2}O$$

(2) $${\mathrm{Cu}}^{2+}+2{\mathrm{CH}}_{3}{\left({\mathrm{CH}}_{2}\right)}_{16}\mathrm{COOH}\to \mathrm{Cu}{\left[{\mathrm{CH}}_{3}{\left({\mathrm{CH}}_{2}\right)}_{16}\mathrm{COOH}\right]}_{2}+2H^{+}$$

Therefore, the copper stearate layer was precipitated on top of the copper substrate with nanostructures.

**Figure 6:**
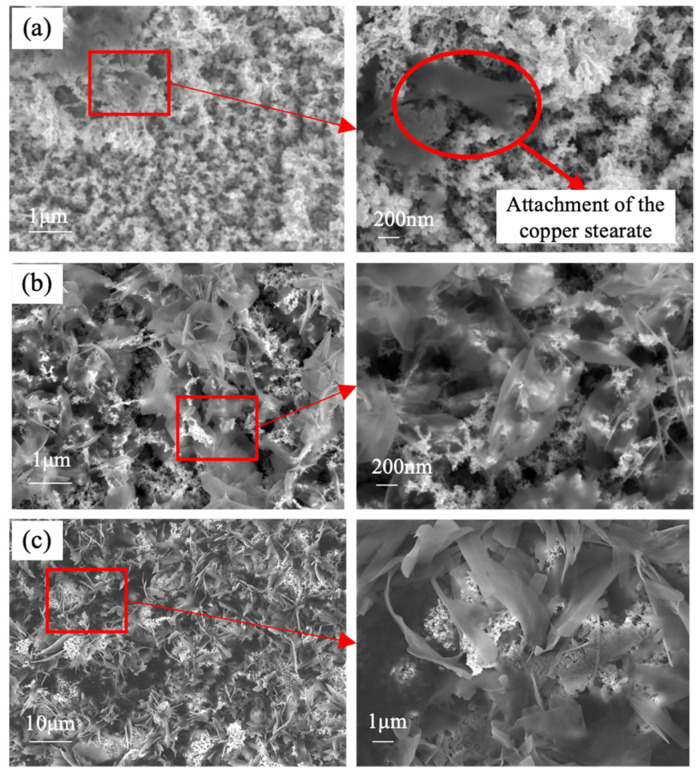
SEM images of the superhydrophobic surface at different modification times: (**a**) 5 min; (**b**) 15 min; (**c**) 120 min.

Figure 6 shows the surface morphology of the superhydrophobic surface during different modification times, including 5, 15, and 120 min. When the modification time was 5 min, a dark color was observed on the copper surface. Through the high-magnification image of this area, we observed copper stearate attached to the nanostructures, and the size of this flaky-like structure was about 1 μm. Subsequently, flakier copper stearates were seen on the surface with a modification time of 15 min, as illustrated in Figure 6b. When extending the modification time to 120 min, the stacked leaf-like copper stearates became thicker and rougher, and they were entangled with each other, nearly covering all the original nanostructures. These copper stearates mainly attached through the pores. This was different from the findings of Wang et al. [21]; they found that flowerlike cluster coatings formed and gathered on a flat copper surface.

### 3.2. Wettability Evaluation of the Superhydrophobic Surface

**Figure 7:**
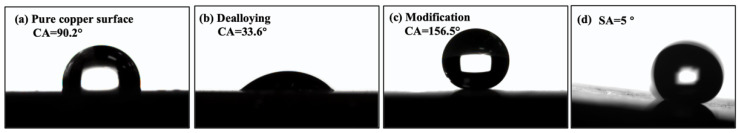
Contact angles of the prepared surface: (**a**) original copper surface; (**b**) dealloyed surface; (**c**) modified surface; (**d**) the water droplet rolling down the superhydrophobic surface and its sliding angle.

The wetting characteristics of the prepared surfaces were evaluated through measuring the contact angle at room temperature. Figure 7 depicts the shape and contact angles of the water droplet on the surface at different stages of the manufacturing process. Initially, the original copper surface exhibited a contact angle of about 90°, as shown in Figure 7a. When the water droplet contacted the surface, the water immediately spread and flowed over the nanopores because of the trapped air, then achieved stability. The contact angle of the surface, exhibiting hydrophilia, decreased from 90.2° (pure copper) to approximately 33.6°. However, after modification treatment, the composite surface contributed to the creation of superhydrophobic properties, and the contact angle reached over 150°. This result could be attributed to the generation of copper stearate, as shown in Figure 6. The pores were blocked and the quantity decreased, preventing the fluid from flowing into the pores. Similarly, Xu et al. [22] prepared a non-flaking superhydrophobic surface with a solution-immersion process on copper foam by means of an ethanolic stearic acid solution (0.05 M) for several days. The surface demonstrated superhydrophobicity with a contact angle of 156°. Li et al. [26] immersed bare copper into an ethanol solution of stearic acid for 72 h, but the contact angle was about 152.3°. Figure 7d shows that the water droplet on the superhydrophobic surface immediately rolled off the surface without any adhesion.

**Figure 8:**
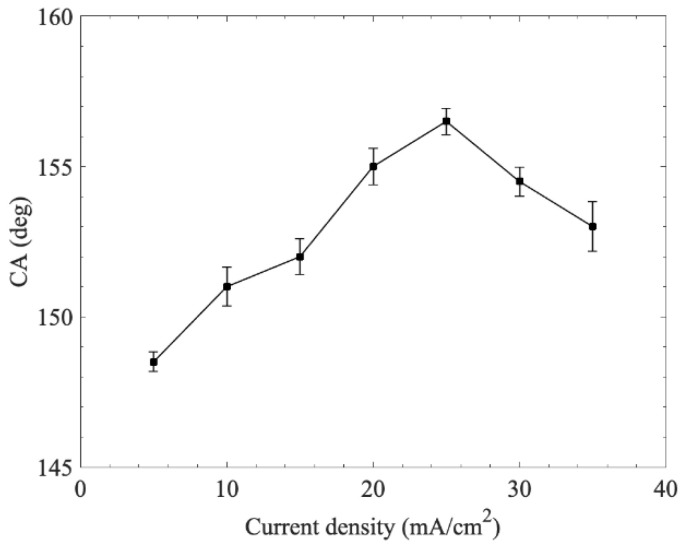
Effect of current density on the wettability of the copper surface.

In this experiment, the thickness of the electrodeposited zine layer was influenced by current, which affected the properties and thickness of the Cu-Zn alloy layer during thermal treatment. Hence, the effect of electric current density was investigated with a modification time of 2 h, as shown in Figure 8. We found that the contact angle for the current density of 5 mA/cm^2^ was 148.3°. The contact angle greatly increased as the current density rose from 5 to 25 mA/cm^2^. After reaching the maximum value, the contact angle rapidly decreased to 152.9° as the current density increased to 35 mA/cm^2^. Thus, to achieve the best superhydrophobic performance, the optimal electroplating current density in the present system was 25 mA/cm^2^, resulting in a contact angle of 156.5°. The reason for this variation tendency may be explained as follows: As the current density increased, the deposited zinc layer on the surface gradually compacted, which was beneficial for the formation of nanostructures. However, when the deposition current was too high, the zinc layer was not compact enough to generate uniform nanostructures within the limited corrosion time [27]. Thus, the resulted nanostructures were coarser compared with those of the low-current sample.

**Figure 9:**
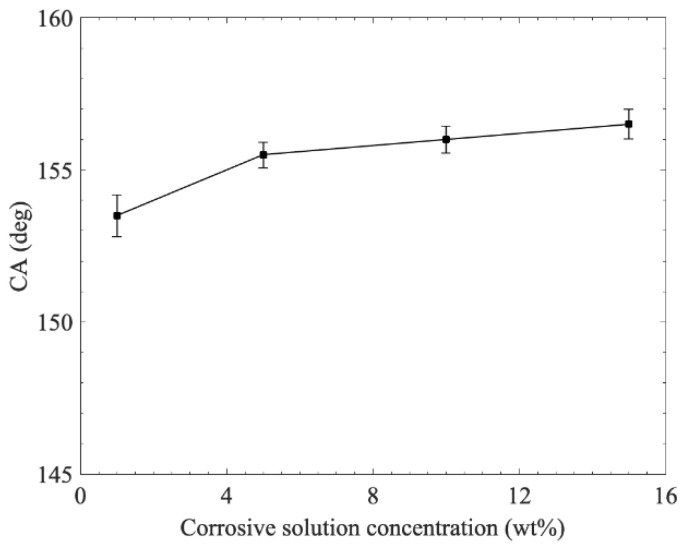
Effect of the corrosive solution concentration on the wettability of the copper surface.

Figure 9 shows the effect of the corrosive solution concentration on the wettability of the copper surface. During this experiment, the current density was consistently at 25 mA/cm^2^, while the modification time was 2 h. The resulting copper surfaces exhibited superhydrophobic performance with different contact angles. As the corrosive solution concentration increased from 1 to 15 wt%, the contact angles showed an upward trend. Particularly, when the solution concentration increased to 15 wt%, the contact angle of the copper surface was 156.5°. This phenomenon may have been caused by the fact that when the solution concentration was sufficiently high, the reaction between H^+^ and Zn was more violent, thereby resulting in the coarser nanostructures on the copper surface [28]. As illustrated in Figure 6c, the coarse surface provided more attachment sites for copper stearate to be generated.

**Figure 10:**
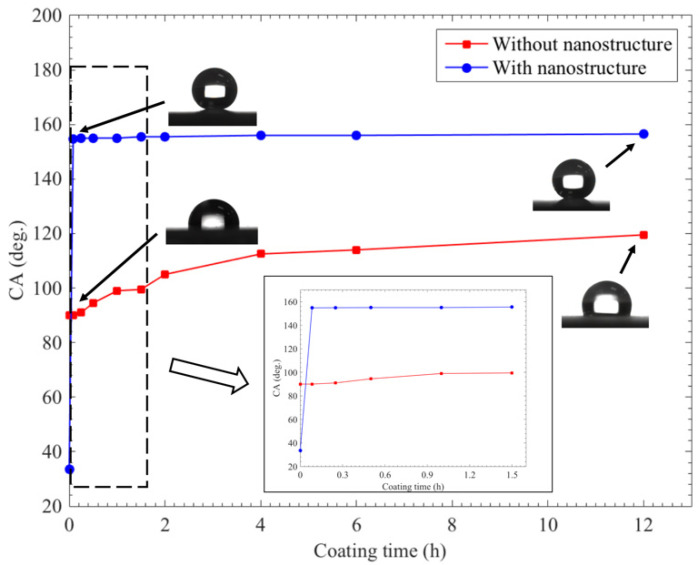
Static contact angles at different modification times (inset shows the contact angles for the coating time between 0 and 1.5 h).

Moreover, the effect of the modification time on the wettability performance is presented in Figure 10. The resulted surfaces presented various levels of hydrophobicity as the modification time rose. During the first 5 min, the contact angle sharply increased from 33.5° to 154.8° (inset of Figure 10). When the modification time exceeded 1 h, the copper surface exhibited superhydrophobic properties. The contact angle sustained the same level of approximately 155°, implying that this method can be used to successfully prepare a superhydrophobic surface. The value was fairly stable, varying from 155.2° to 156.7° during a coating time of 12 h. In order to better understand the effect of the dealloying method on nanostructures, we carefully compared the changes in wettability for bare copper surfaces. During this stage, the copper surface was polished with SiC paper down to 2000 grit, then immersed into a 0.005 mol/L ethanol solution of stearic acid. The results of the contact angle are shown in Figure 10 (red curve). The contact angle showed a slightly upward trend within the measurement. A contact angle of 94.4° was achieved in 30 min, and the value increased to 119.6° after modification for 12 h. This result was consistent with the findings of Wang et al. [21]. They found that the coverage of copper substrates could not become superhydrophobic unless the formation of the copper stearate clusters was dense enough. Hence, the contact angle could not surpass 120° by the end of the test (after a modification time of 12 h).

Therefore, the wetting behavior became superhydrophobic, owing to the fact that the nanostructures of the copper surface were superior to those of the copper substrate. These differences may have been caused by two factors: the nanostructures provided an adequate area for chemical reactions to occur between copper ions and the stearic acid solution. According to the Cassie model on the wettability of rough surfaces, liquid droplets cannot easily sink into small cavities. The trapped air in the cavities, supported by micro- and nano-structures, prevents liquid from entering the pores. The water contact angle according to the Cassie model can be expressed as,

(3) $$\cos\left(\theta_{c}\right)=f_{1}\cos\left(\theta_{e}\right)-f_{2}$$

(4) $$f_{1}+f_{2}=1$$

where $\theta_{c}$ and $\theta_{e}$ are the water contact angles of the rough and smooth surfaces, respectively. $f_{1}$ and $f_{2}$ are the fractional areas estimated for the solid and air contact points with the surface, respectively [29]. In this study, for the surface with nanostructures, the contact angles of the prepared copper surface with stearic acid modification and a smooth surface were $\theta_{c}$ = 156.5° and $\theta_{e}$ = 90.2°, respectively, as shown in Figure 7. The calculated $f_{1}$ and $f_{2}$ were 0.083 and 0.917, respectively. The results indicated that the contact area between the water droplet and the copper surface was merely 8.3%, while that of the air-occupied fraction exceeded 91.7%. In comparison, the contact areas for the copper surface without nanostructures was calculated to be $f_{1}^{\prime }$ = 0.507 and $f_{2}^{\prime }$ = 0.493. The contact area with the water droplet was 50.7%. The nano- and micro-pores showed a potential to receive the leaf-like copper stearate clusters. As shown in Figure 6, one end of the leaf-like structure was fixed inside the pores, thus preventing adhesion. Hence, because of the existence of nanostructures, the hierarchical micro- and nano-architectures facilitated the formation of a copper surface with superhydrophobic performance.

### 3.3. Applications of the Superhydrophobic Surface

**Figure 11:**
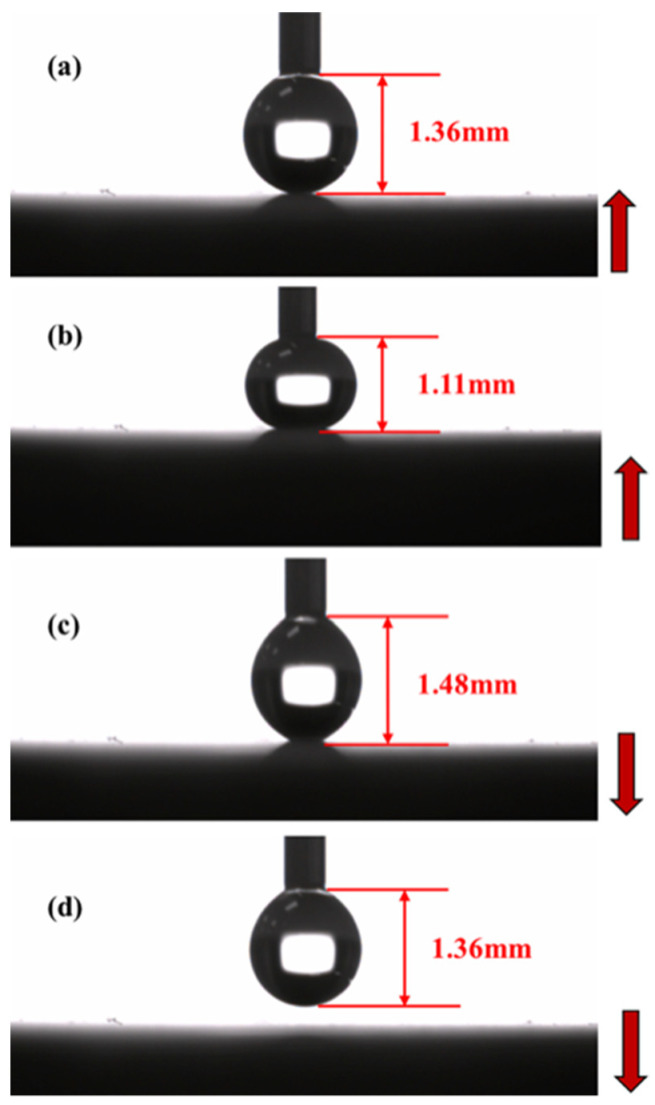
Photographs of the contact and departure process of water droplet (**a**) slightly contacted with the superhydrophobic surface, (**b**) compressed by the surface, (**c**) stretched by the moving surface, and (**d**) departed from the surface. The arrows show the movement directions of the surface.

For practical applications, we conducted experiments to evaluate the non-sticking property of the superhydrophobic surface through the “contact and departure process” according to the method outlined by McCarthy [30,31,32,33]. As shown in Figure 11, a 4 μL water droplet was first suspended beneath a needle (Figure 11a). As the surface elevated, the length of the droplet compressed from 1.36 to 1.1 mm (Figure 11b). Despite this, the droplet retained a somewhat spherical shape. When the surface lowered, the length of the droplet extended 1.48 mm (Figure 11c). However, the viscosity force was not sufficient to pull down the water. When the water droplet departed from the superhydrophobic surface, it quickly returned to its original shape (Figure 11d). Under normal conditions, this “contact and departure” process occurred several times during the test, indicating that the superhydrophobic surface possessed non-sticking properties.

**Figure 12:**
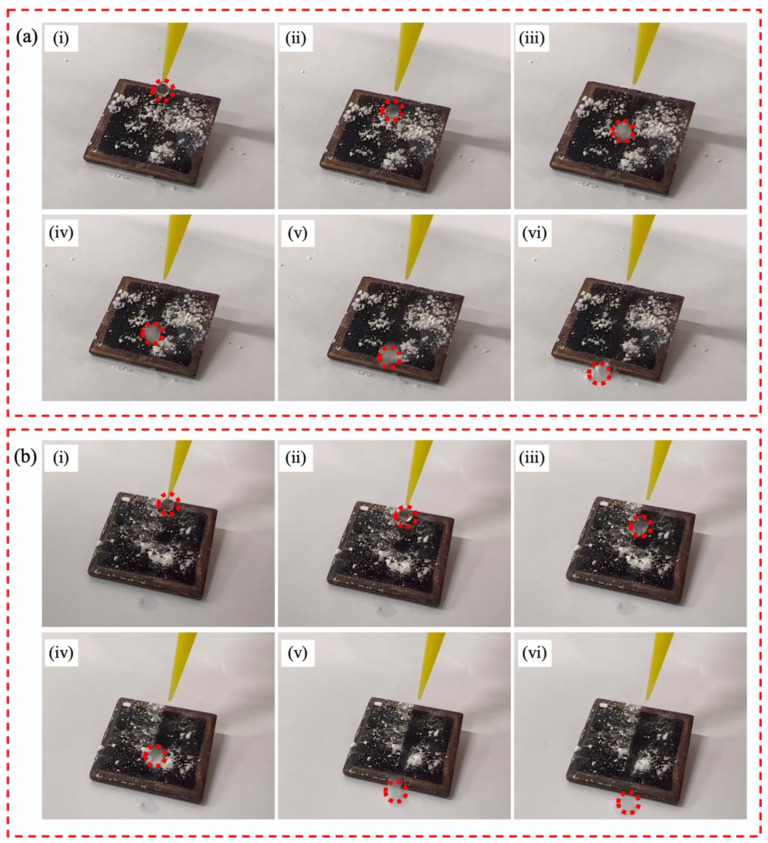
Self-cleaning process of the superhydrophobic surface: (**a**) inclination angle of 8°; (**b**) inclination angle of 20°. Red circle implies the water droplet.

The self-cleaning performance of the superhydrophobic surface was also investigated in this study. As shown in Figure 12, a layer of sand was first sprinkled on the superhydrophobic surface. Then, the sample was positioned at an inclination angle of 8° (Figure 12a) and 20° (Figure 12b). The water droplet was dribbled onto the surface. As the water droplet rolled, it immediately and easily removed the sand. Consequently, the surface was cleared as several droplets flowed along the superhydrophobic surface. This phenomenon indicated that the copper surface possessed a superior self-cleaning property.

**Figure 13:**
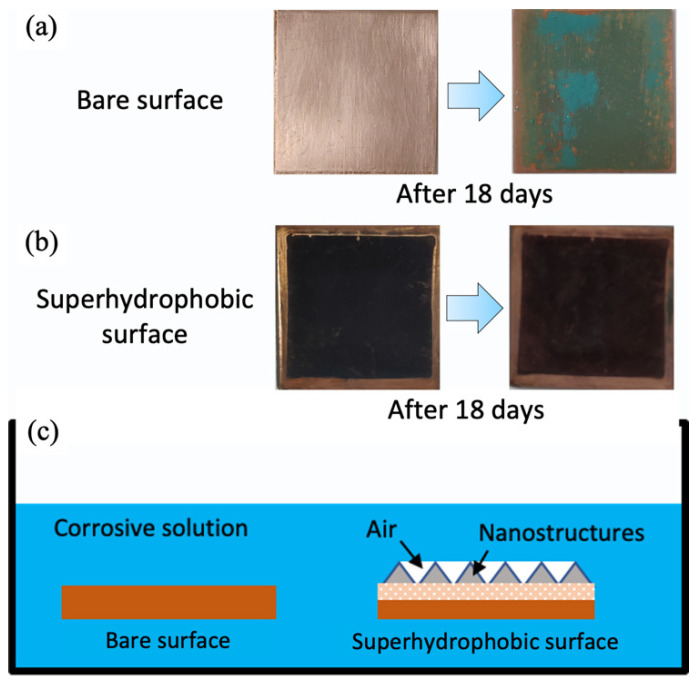
The optical images of the bare copper surface and superhydrophobic surface (**a**) before and (**b**) after immersion into a 5 wt% NaCl solution; and (**c**) anti-corrosion mechanism of the superhydrophobic surface.

Furthermore, the anti-corrosion performance of the superhydrophobic surface was investigated through immersing it into a 5 wt% NaCl solution. Before the anti-corrosion test, the bare copper substrate presented a bright color. However, after being immersed in the NaCl solution for 18 days, the copper surface was covered in patina, indicating that the surface was directly corroded, as shown in Figure 13a. In comparison, the superhydrophobic surface retained its black shade throughout the entire anti-corrosion test, implying that the as-prepared surface had relatively superior corrosion resistance in the above-mentioned corrosive media, as shown in Figure 13b. Figure 13c shows the anti-corrosion mechanism of the superhydrophobic surface in corrosive solutions. Regarding the bare copper surface, the water and corrosive ions in the corrosive solution could directly contact the copper surface, thus accelerating the corrosion rate. However, for the superhydrophobic surface, copper stearate constructed hierarchical structures on the substrate, and air was trapped in these “valley” structures. Therefore, the trapped air effectively prevented the penetration of the NaCl solution and reduced the corrosion rate. However, this method may not be appropriate with other metals. For example, Kulinich et al. [34,35] coated smooth aluminum surfaces with stearic acid. They found that upon long-term immersion in water, the aluminum surface with alkyl groups tended to gradually decay, which was caused by reactions with water.

**Figure 14:**
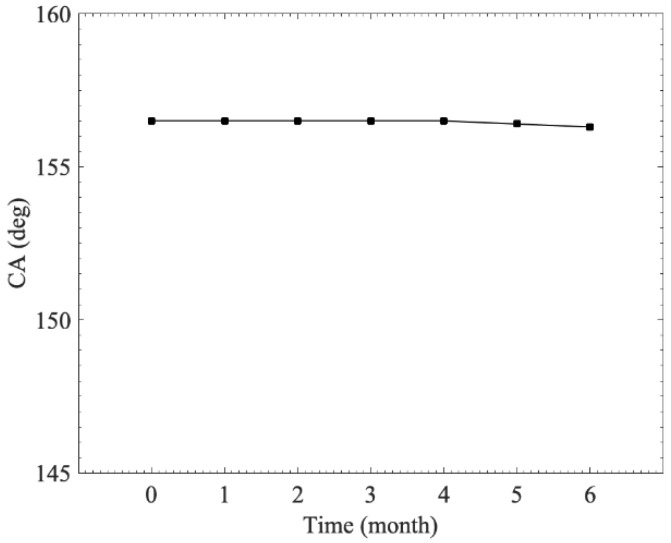
Stability of the superhydrophobic surface.

We considered stability to be a key parameter for practical applications. We evaluated the stability of the superhydrophobic surface by measuring contact-angle variation according to the time spent at room temperature. Figure 14 shows the wetting behavior of the superhydrophobic surface. The contact angle of the treated surface remained in a superhydrophobic state after 6 months. Thus, the as-prepared sample was stable enough to be employed long-term under environmental conditions.

## 4. Conclusions

Nanostructures were prepared on a copper substrate by an alloying and dealloying method, then a superhydrophobic surface was produced through immersion into an ethanol solution of stearic acid. The morphological and component analyses of the fabrication process were conducted on the treated surface with SEM, XRD, and FTIR. The effects of the electric current density, corrosive solution concentration, and nanostructures were analyzed and compared to optimize the fabrication process. The results showed that after the alloying and dealloying treatment, the copper surface was covered with nanostructures, exhibiting hydrophilicity. However, it became superhydrophobic after modification in an ethanol solution of stearic acid. The nanostructures on the copper surface could effectively facilitate the attachment of the low-surface-energy leaf-like copper stearate components. The optimal electroplating current density and corrosive solution concentration in the present system to achieve the best superhydrophobic performance were 25 mA/cm^2^ and 15 wt%, respectively. A superhydrophobic property could be imparted to the copper surface within a modification time of 5 min. Meanwhile, the formation of the hierarchical structures, with long-term stability, yielded significant non-sticking behavior, good self-cleaning, and anti-corrosion properties. Therefore, this method can be utilized for industrial applications in the field of superhydrophobic surface fabrication.
